# Social and political determinants of health in the occupied Palestine territory (oPt) during the COVID-19 pandemic: who is responsible?

**DOI:** 10.1136/bmjgh-2020-003683

**Published:** 2020-09-25

**Authors:** Weeam Hammoudeh, Hanna Kienzler, Kristen Meagher, Rita Giacaman

**Affiliations:** 1 Institute of Community and Public Health, Birzeit University, Bir Zayt, West Bank, Palestine, State of; 2 Department of Global Health & Social Medicine, King's College London, London, UK; 3 War Studies, King's College London, London, UK

**Keywords:** health policy, health systems

**Summary box**

- Israeli military occupation of the Palestinian territory has resulted in stunted development, weak and underfunded health and social services.
- The structural predicament in the occupied Palestinian territory (oPt) has exacerbated political, economic and social instability during the COVID-19 outbreak.
- The current situation cannot be detached from the broader context of settler colonialism and the logic of colonisation.
- Several groups are acutely disadvantaged and marginalised by the COVID-19 restrictions, including women and young people.
- Mental health is often overlooked in this context and psychosocial support is often unavailable.
- The lack of long-term development exacerbates the current situation and questions the role of humanitarian and development aid.
- By questioning responsibility, the COVID-19 pandemic in the oPt may represent an opportunity for justice and freedom for Palestinians.

## Background

The Palestinian authority (PA) reacted quickly to the coronavirus outbreak, introducing strict lock down measures to limit community transmission. However, since restriction measures were eased in late May, there has been a massive spike in cases with over 9000 cases by July 18 2020 compared with less than 400 cases at the end of the lockdown. This has created a climate of fear. Since the spike, shorter lockdown measures have been instituted, but have been met with protests due to the lack of economic protection for the population. Going forward, long-term lockdown measures are likely unable to be implemented in the occupied Palestinian territory (oPt) as the financial and social consequences have had devastating impacts on the nation as a whole.

## Why are the impacts of COVID-19 exacerbated in the oPt?

Israeli military occupation of the West Bank and Gaza Strip has lasted over 50 years, with lack of access to land, water, borders and the freedom of movement of people and goods from one part to another and even within parts of the country. This has resulted in stunted development, also known as dedevelopment, weak and underfunded health and social services. In the wake of the COVID-19 outbreak, this structural predicament has heightened political, economic and social instability.

During recent months, there has been an increase in targeted attacks by Israel, exacerbating an already fragile political and economic situation. This includes blockade and attacks on the Gaza Strip, demolition of homes and attacks on infrastructure including healthcare facilities in the oPt, and the continued annexation of land. In addition, the COVID-19 pandemic is compounded by an already resource scarce setting with a weak health infrastructure. While problematic in itself, the current situation cannot be detached from the broader context of settler colonialism and the logic of colonisation. This has resulted in a double epidemic in the oPt. While the Palestinian Ministry of Health (PMoH) has done the best it can with limited resources, the oPt continues to operate in ‘emergency mode’. Furthermore, the current political context limits the PA’s jurisdiction and control over borders. This has played a key role in securing personal protective equipment and testing supplies, which have been in short supply at different points and possibly part of the explanation for why the sharp increase occurred where an increase in cases largely went undetected as people continued to interact as measures eased. The lack of control over borders and lack of sovereignty in areas classified as B and C, particularly since coordination with Israel has been stopped, have limited the PA’s ability to manage the situation and assure implementation of prevention guidelines in various areas.

## COVID-19 and mental health

The stunted, under-resourced and fragmented health system drives the climate of uncertainty in accessing adequate health services and fuels wider uncertainty stemming from the political situation. The uncertainty associated with the pandemic has been particularly burdensome for Palestinians on two levels. First, economically, as there are no social protection measures available to support persons and families. Second, socially, as people were asked to stay physically away from their social support network—a foreign concept for Palestinians, who in times of political crises have drawn heavily on social solidarity.

The uncertainty, compounded by lockdown measures, impacts everyone. However, several groups are acutely disadvantaged and marginalised by the COVID-19 restrictions. The gendered impact of COVID-19 has seen women burdened with additional home and caring responsibilities and a possible increase in domestic violence. Young people (18–22) are unable to plan for the future. Workers in the informal sector have also been severely impacted with no alternatives to provide for themselves or their families. Persons with disabilities and their caretakers are constrained by a lack of adequate health and other support services.

Mental health is also often overlooked in humanitarian settings and psychosocial support is often unavailable. The Palestinian Counselling Centre (PCC) (2020) conducted a rapid review and needs assessment of mental health and psychosocial support needs of people in the West Bank (https://www.pcc-jer.org/en/content/rapid-assessment-needs-14-marginalized-areas-west-bank-during-covid-19-epidemicee). Key findings were that people’s mental health and well-being were adversely affected by an increase in Israeli military occupation-related violence (raids and arrests) during the lockdown measures imposed by the PA to deal with the pandemic; unemployment and poverty as people’s livelihoods were at stake due to lockdown and social distancing measures; overcrowding in houses; and lack of water and sanitation in some areas. Such pressures were linked to increased worries, anxieties and tension in families and in some cases domestic violence. Unfortunately, no information is available about how people with severe mental health issues are coping during this time. Our own research has shown how, at the best of times, their lives are challenging, precarious and often insecure. With regard to mental health and psychosocial support the report noted that in many areas, especially in area C, there was little to no mental health and psychosocial support available. In other regions there would have been mental health professionals and counselling students willing to volunteer—but, their services were not included in the emergency response. The PCC recommends involving mental health professionals and student volunteers in the provision of phone counselling to improve the response; creating recreational activities/spaces for young people; easing economic pressures for poor families through donations and providing protection from occupation-related violence.

## The role of humanitarianism and aid

The lack of long-term development further exacerbates this situation, questioning the role of humanitarian and development aid. Humanitarian interventions, while important in a war-affected setting, do not come without costs as they have been shown to hamper long-term development considering that their visions and outcomes are treated as projects with short timelines and narrow goals, rather than systemic programmes impacting the livelihoods of people. The benefits of delivering healthcare in the form of projects are twofold. First, projects tend to be flexible for donors while binding recipients of aid to sometimes erratically changing timelines and project aims; have narrow set goals; and predefined end dates often no longer than 1–2 years. International donors and project managers can, therefore, react flexibly and more effectively to changes in administration, funding interruption and political climate without committing to development goals. While this fits the funders, it makes the work of aid recipients extremely challenging as they act as a distraction from systemic oppression. Second, the delivery of healthcare in the form of projects allows international actors to provide aid without questioning the status quo of Israeli occupation and involvement of Europe and the USA in the quagmire; that is, it allows aid provision without laying bare and questioning historical injustices to address the root causes of the Palestine Question and develop long-term, sustainable development of the health sector. The pandemic and its response has raised once more important questions about how do we move the conversation from being merely donor pleasing to building sustainable systems that are locally responsive and include the most vulnerable? How do we openly shift the conversation to challenge the root causes of why the health system is weak and fragmented? This brings into question who is responsible for not only Palestinians accessing health services during COVID-19, but who is responsible for the justice and freedom of all Palestinians.

## Who is responsible?

The limitations in the Palestinian health services’ abilities to fully cope with the pandemic are intertwined with the above outlined structural and political determinants. What this situation has also shown is that an approach to the health system needs to focus on long-term development, sustainability and preparedness rather than solely on humanitarian and emergency assistance that are short-term band aids. Yet, considering that development is supposed to take place within the context of military occupation, it begs the question: Who is in fact responsible for guaranteeing the health of the Palestinians under occupation during the pandemic and beyond?

The PMoH is the main provider of healthcare services in the oPt, followed by United Nations Relief and Works Agency (UNRWA), which caters to the needs of Palestinian refugees of the 1948 and 1967 wars, and a range of non-governmental organisations. However, under the Geneva Conventions, Israel as the occupying power, is responsible for the health of the population, or at the very least facilitating access to healthcare services. The current situation in the oPt only intensifies the question of responsibility. It is not simply a question of responsibility and the right to health during COVID-19. Responsibility of the injustices and freedom of Palestinians has continually been overlooked by not only the occupying power, but the international community. It illuminates the issue of focusing on those disadvantaged and the distribution of aid rather than long-term, system development. By focusing on this disadvantage, we fail to question and dismantle unjust advantage in order to address root causes and question the advantage of (external) communities and humanitarianism is used to conveniently disregard the wider, intrinsic root causes of injustice. How can COVID-19 be framed as an opportunity for justice and freedom for Palestinians, which makes global powers accountable, and gives a voice to the Palestinian people on asserting Palestinian rights?

